# Estimation of the Spacing Factor Based on Air Pore Distribution Parameters in Air-Entrained Concrete

**DOI:** 10.3390/ma18081716

**Published:** 2025-04-09

**Authors:** Jerzy Wawrzeńczyk, Henryk Kowalczyk

**Affiliations:** Faculty of Civil Engineering and Architecture, Kielce University of Technology, Al. Tysiąclecia Państwa Polskiego 7, 25-314 Kielce, Poland; zmsjw@tu.kielce.pl

**Keywords:** air-entrained concrete, air-void system, Powers model, spacing factor, artificial neural network

## Abstract

Air-void characteristics are defined in the EN-480-11 test method. The primary criticism of Powers’ model comes from the fact that the spacing factor is calculated with the average chord length, without taking into account the chord length distribution. The aim of this study is to determine whether an analysis of the chord length distribution can provide a more accurate estimate of the spacing factor. A data set containing 110 air-entrained concretes with various characteristics was analyzed. The artificial neural network method was applied to develop a model that determines the relationship between the spacing factor, L2, and the parameters of the air-void structure. The input parameters for the ANN-L2 model included the following: A, d, and W—characteristics of the chord size distribution, P—cement paste content, and N5—number of large pores. The ANN model allows for a sufficiently accurate estimation of the spacing factor, L2. The most significant factors that influenced L were the peak amplitude, A; peak width, W; and cement paste content, P. There was a strong correlation between the results of the ANN model and the standard spacing factor L2, indicating that both calculation methods produced comparable results. Finally, a simple method for using the ANN model to calculate the spacing factor in Excel is demonstrated.

## 1. Introduction

The number and distribution of air void sizes are crucial characteristics that significantly influence the air-pore system, which protects the concrete from cyclic freezing and thawing [1,2].

Traditionally, the spacing factor proposed by Powers [3] has been considered an appropriate metric to describe the structure of air voids. Powers introduced a remarkably innovative but simple model to calculate the spacing factor (Table 1), relying on relatively straightforward microscopic measurements.

The quality of the air-void system in concrete is quantitatively assessed based on the following three key parameters: air content (A), specific surface area (α), and spacing factor (L). The methodology for measuring these parameters is described in standards such as the ASTM C 457 [4] and EN 480-11 [5].

Although this method has been used for over 70 years, challenges related to proper air-entrainment and quality control remain unresolved. Discussions surrounding this topic occur on multiple levels, as follows:

- Theoretical considerations regarding the best way to describe the air-pore system;
- Measurement methodology, including whether to use linear, surface, or spatial techniques, as well as manual or automated image analysis;
- Sample preparation techniques, such as contrast enhancement or extraction of air voids from the background in flat microsections, as well as natural surface analysis or 3D imaging without sample preparation;
- The relationship between the spacing factor and the cyclic freezing and thawing results.

The overall goal of these efforts is to develop a method utilizing automated image analysis to accurately detect even the smallest pores in concrete, determining the pore size distribution in 3D space. Additionally, it will aid in identifying an easily measurable air-void parameter that strongly correlates with freeze–thaw damage.

The foundation of the Powers method is the specific surface area of the voids, which is directly related to the average length of the measured chords. It is widely accepted that only a portion of air voids contribute to the frost resistance of the concrete. Air voids in air-entrained concrete vary in size. They include desirable pores (d = 10 ÷ 100 µm), which form because of air-entraining admixtures, as well as larger entrapped air voids (d > 1 mm) caused by inadequate compaction. The parameter (A300) that distinguishes useful pores from nonfunctional ones concerning frost resistance is the micropore content of the air voids with diameters up to 300 µm [6]. In the US, pores up to 1 mm are considered useful. Larger pores are believed to have no effect on frost resistance but merely increase the overall porosity of the concrete.

When entrapped air voids are excluded from calculations, the spacing factor value typically decreases [7,8,9]. Pigeon and Pleau [1] argue that the presence of just a few large air voids can significantly increase the mean pore diameter. This reduces the specific surface area and, consequently, increases the spacing factor value. The spacing factor may decrease from 300 µm to 200 µm when large air voids are omitted [1]. Walker [8] proposed a spacing factor based solely on smaller entrained-air voids and developed an expression for the small spacing factor.

A key advancement in EN 480-11 was the introduction of a method for estimating the 3D pore size distribution using the 1D chord length distribution divided into 28 length classes. This approach enables the estimation of the micropore content below 300 µm (A300). The calculations are based on the Schwartz–Saltykov method [10,11]. Later, a simplified method for determining the A300 parameter was proposed by Wawrzeńczyk and Kowalczyk [12]. However, the air-void distribution has no other practical application.

Snyder et al. [13] analyzed the influence of the number of voids and the length of the traverse on the accuracy of the results in a linear traverse method. They suggest that the desired number of chords should be about 1000 pores. Additionally, the accuracy is only marginally improved when analyzing more than 1000 pores. Virtually no improvement occurs beyond 2000 pores. If the number of pores is very low due to low air content or the presence of large pores, the traverse length recommended in ASTM C 457 may be insufficient for obtaining accurate air-void parameters. The authors suggest extending the traverse length to ensure precision.

Criticism of Powers method arises from its reliance on a single parameter, the average chord length—which strongly influences the spacing factor. Many experts have argued that the calculations should account for the actual chord length distribution. Various alternative approaches have been proposed, including those by Philleo [14], Attiogbe [15], Elsen et al. [16], Mayercsik et al. [17], and Pleau et al. [18].

According to Philleo, each air bubble protects the surrounding cement paste within width S from frost damage. It is the random distribution of air voids, rather than their total volume in the paste, that plays a crucial role. The spacing factor, S, proposed by Philleo is based on a logarithmic distribution.

Attiogbe used a gamma distribution to define the F parameter, which, like S, represents the volume of paste potentially protected by air bubbles. Unlike the standard factor L, both S and F account for the random nature of the size distribution and spacing of air voids, as well as their actual number. The shortcomings in estimating the spacing factor L result in the lack of a consistent correlation between L and the freeze–thaw resistance results. The conclusions drawn from the analyses by Attiogbe [15] indicate that parameter F correlates better with freeze–thaw test results compared to the standard spacing factor L.

Philleo used the total number of voids per unit volume to calculate the spacing factor. Other researchers have attempted to use the chord length distribution curve to represent air-void spacing [19]. They suggest that the pore size distribution should be considered to fully characterize the dispersion of air voids in concrete.

Lord and Willis [20] suggested that a spacing factor based on the average values of the actual void system may be more effective than one based on the total specific surface. Possible examples included the actual mean void diameter, the diameter of a sphere with the average volume, or the diameter of a sphere with the average specific surface. To determine these average void measures, knowledge of the volumetric size distribution of air voids is necessary. They developed a graphical method to determine the volumetric size distribution based on the measured distribution of void chord lengths obtained from random linear traverses.

Larson et al. [21] used an exponential function to fit the measured chord length distribution. Roberts and Scheiner [22] used a logarithmic distribution to describe the measured chord distribution.

Advancements in electronics and computational methods have enabled the use of automated image analysis (AIA) to characterize concrete air-void structures. AIA offers a key advantage by providing the size distribution of circular voids intersected by the surface of the examined concrete sample. This distribution more accurately reflects the true air-void size distribution within cement paste than the chord length distribution obtained from the linear traverse method described in ASTM C 457 [1].

The development of automated image analysis has allowed for the extraction of data from polished concrete sections using cross-sectional analysis (2D). Today, such tests can be routinely performed using systems such as RapidAir [23]. Researchers, including Pleau et al. [18], Mayercsik et al. [17], Zalocha and Kasperkiewicz [24], Soroushian et al. [25], and Aligizaki et al. [26,27] as well as Wawrzeńczyk and Kozak [28], have applied 2D analysis in their studies.

Numerous studies have aimed to reduce manual analysis by integrating color-based image segmentation with advanced image processing techniques [29]. Early automated methods relied on mechanical stage microscopes to capture images of polished concrete surfaces. An alternative approach, flatbed scanning, has gained popularity because of its cost-effectiveness in detecting concrete voids [24,30].

Despite these advantages, color-based contrast enhancement has inherent limitations, as follows: (i) color thresholds must be manually adjusted for each experiment due to subtle variations in sample preparation; (ii) void detection and segmentation accuracy depend heavily on the type, particle size, and compatibility of color treatments [31].

To overcome these limitations, more advanced methods have emerged that do not rely on the enhancement of the black-and-white contrast of polished sections. Dedicated algorithms now detect air voids by analyzing the shadows cast under oblique illumination. Song et al. [31] employed spectral analysis to segment scanned RGB images, using phenolphthalein and fluorescent chalk powder to distinguish between phases. The open-source software Multispec was used to classify air voids, aggregates, and paste phases [32].

The HF-MAC01 device [33] further enhances shadow detection by illuminating specimens from multiple angles. A similar approach has been adopted by other researchers [29]. A notable development is the NG-ACE method [34], which employs neural networks pretrained to recognize the specific color features of each component. This technique generates a color map correlated with the identified void structures. Neural networks for air-void detection are also integrated into newer versions of the Nikon Imaging System (NIS). Other studies have applied fractal analysis methods [35,36].

There is growing interest in investigating the air-void size distribution in concrete using 3D X-ray computed tomography (CT) [37,38,39,40,41]. The CT method offers the following significant advantages: (i) it does not require extensive sample preparation and (ii) it enables direct measurement of actual pore diameters in three-dimensional space.

Recent studies indicate that modern air-entrained concretes contain a higher proportion of smaller pores than those produced with older air-entraining admixtures, such as Vinsol resin (Pleau et al. [42]). Accurate measurement of these small voids (≈10 µm) is crucial for reliable L-factor calculations. However, the resolution of 3D CT imaging remains a limiting factor, as current voxel sizes range from 30 to 40 µm. This may be insufficient to capture the smallest air voids. As a result, the data obtained using this method should be interpreted with caution. Despite advancements in technology, CT-based automated methods are still not widely feasible for analyzing real-world concrete samples, mainly due to specimen size and resolution limitations. This prevents their adoption as routine quality control tools in construction [29].

Although various approaches have been proposed to assess air-void structures in concrete, the standard method [4,5] is still commonly used. It involves the measurement of chord lengths via microscopic examinations and the parameters are then calculated using Powers’ equations.

The authors of [12] presented an analysis of an extensive data set containing information on 290 pavement concretes with highly diverse air-void structures. The aim of the analysis was to develop a simpler method for estimating the A300 parameter without performing the tedious calculations described in EN 480-11. The developed function includes only a few coefficients (eight classes) because only chord lengths in the range of 10–350 µm are statistically significant. Moreover, the presented approach may have consequences for the methodology of testing parameters characterizing the structure of air-entrained concrete using the 2D method. The conclusions drawn from the analysis are as follows:

- Chords with lengths of 0 ÷ 10 µm and greater than 355 µm have no statistically significant effect on the A300 value;
- Only eight classes within the chord size range of 15–350 µm are statistically significant.

The class (305–350 µm) negatively affects the A300 value. This is evident, as chords of this length are too large to be included in the A300 parameter. An increase in the number of chords in this class reduces the A300 value. This is because these chords result from a transverse intersection cutting through much larger pores.

Considering this, the following question arises: should larger pores be included in the microscopic analysis at all?

This study focused on determining the void distribution parameters based on chord length measurements. The authors are not aware of any example in the literature of a direct application of air-void distribution parameters to the calculation of the spacing factor L.

The primary objective was to answer the following fundamental question: Does incorporating characteristic parameters of the chord length distribution allow for a more accurate determination of the spacing factor value compared to the standard method based on the average chord length? An attempt to solve this problem consisted of developing a model defining the relationship between the spacing factor (L2) and the parameters of the air structure. The artificial neural network (ANN) method was used as a multivariate regression analysis technique.

## 2. Materials and Methods

In Reference [12], an analysis is presented of a data set containing the results of microscopic measurements of pavement concretes. The collected data set contains parameters determined for 292 concretes. These parameters include air content (A), micropore content (A300), specific surface area (α), and number of chords (N) within a chord length range of 0 ÷ 4000 μm. Recently, the data set was expanded to include the results from an additional 40 concretes. These parameters were determined using the Rosival linear traverse method, in accordance with the EN-480-11 standard, which involves counting the chord lengths. Microscopic measurements were performed using the NIS-Elements AR 4.6 software, which records individual chord lengths and enables data to be exported to a spreadsheet (MS Excel). When additional input data are provided, such as the paste volume (P) and total traverse length (Ttot), a custom VBA macro is used. This macro processes the data according to the EN-480-11 standard.

Typically, the number of measured chords in concrete, as specified by EN-480-11, ranges from 400 to 1200.

**Figure 1:**
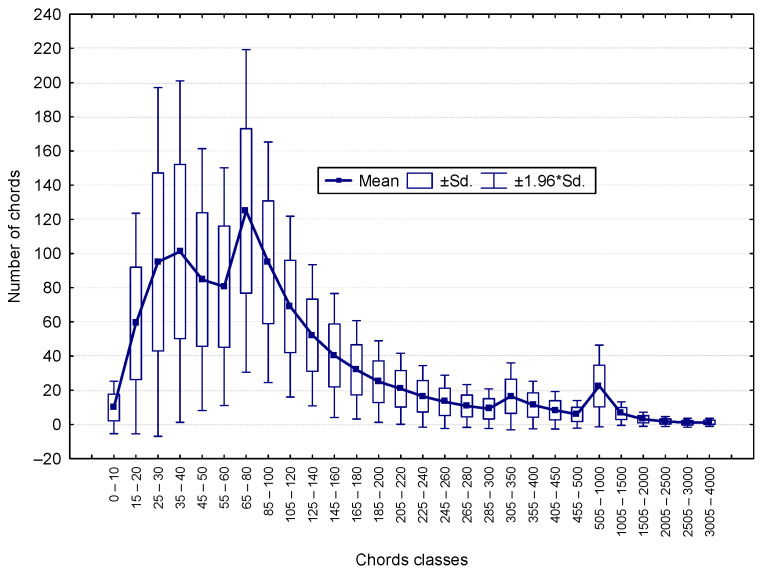
Characteristics of the distribution of the number of chords in each class [12].

When analyzing the distribution of chord dimensions in concrete (Figure 1), it can be observed that this distribution is not continuous. Certain classes contain a high frequency of chords, while others show little to no presence of chords. The distribution of chord lengths across 28 classes, as depicted in Figure 1, reveals a significantly distorted representation of the true distribution. Specifically, as shown in the figure, most of the chords are concentrated within a range below 250 µm. Their number decreases as the chord length increases.

Two primary peaks can be identified in the chord length distribution, as follows:

- The first peak spans the range of 10 ÷ 500 µm;
- The second peak spans the range of 500 ÷ 1000 µm.

**Figure 2:**
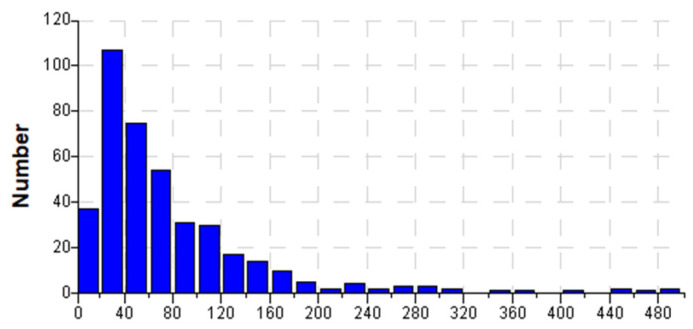
Histogram of the number of chords across the selected 25 classes.

For the purposes of this analysis, a subset of 110 concretes (i.e., records) was selected. The chord length distribution was analyzed across 25 classes, each with an interval width of 20 µm, covering a total range of 0 – 500 µm. The NIS AR 4.6 software [43] enables the generation of a table (Table 2) summarizing the frequency of chords across the selected 25 classes, as well as a histogram (Figure 2) illustrating the distribution.

The further analysis aimed to determine the characteristic parameters of the chord length distributions. Specialized software, TableCurve 2.0 [44] was used, allowing for the fitting of various distribution models. For each concrete sample, the best-fit distribution was determined based on the lowest estimation error or the highest determination coefficient (R^2^). Based on the analysis conducted, the distributions that best matched the measured data were identified as lognormal, gamma, inverse gamma (InvGamma), and extreme value (ExtVal). The Weibull distribution frequently ranked next.

**Figure 3:**
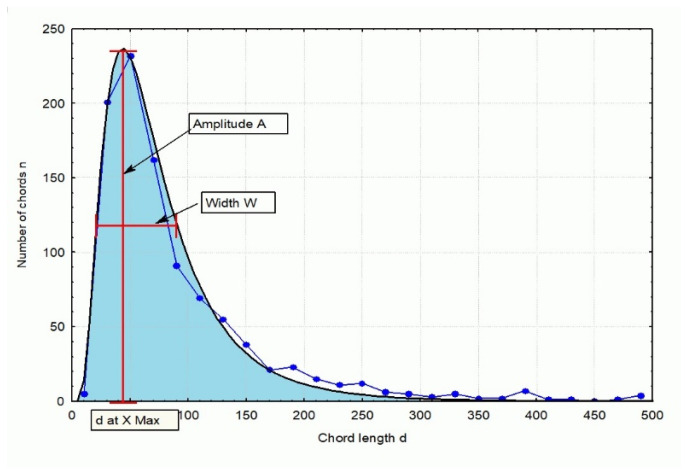
Parameters characterizing a given chord distribution.

Each distribution can be characterized using three key parameters (Figure 3), as follows:

- Peak amplitude (A);
- Chord length (d) corresponding to the maximum peak value;
- Peak width (W) at half of the peak’s maximum height.

In a subsequent stage of the study, an additional class within the range of 500–1000 µm was included, for which the number of chords (N5) was determined.

Table 3 presents information characterizing the variability of parameters related to the composition and distribution of individual concretes. The result of these analyses was a data set containing 110 records, each consisting of six values corresponding to the data listed in Table 3.

## 3. Model ANN-L2

The primary objective of the presented analysis was to develop a numerical model. This model should be capable of estimating the spacing factor (L) based on chord length distribution parameters (A, d, W). The estimation is supplemented by the number of chords (N5) in the 500–1000 µm range and the cement paste content in the concrete (P). The task involved determining a regression relationship between the input factors and the target output variable, which is the spacing factor L2 (where only chords shorter than 2000 µm are considered). To solve this problem, the artificial neural network (ANN) method was applied. Neural networks represent a modern and advanced modeling technique capable of capturing highly complex nonlinear relationships. Unlike traditional statistical methods, they do not require the user to formulate complex hypotheses about the form of statistical dependencies, while also providing effective control over the multidimensional nature of the problem [45]. It is worth noting that the adopted approach enables the inclusion of information about chord frequency across 26 classes using only three distribution parameters (A, d, W).

The analysis encompassed a data set containing 110 records, each consisting of six values, corresponding to the data provided in Table 3.

The calculations were performed using QNET’97 software [46]. Neural networks utilizing the backpropagation algorithm require that all training target values be normalized to the range of 0–1, as the output node signals are constrained within this range. QNET also requires input normalization to enhance the training performance. The software automatically normalizes the data, scaling all input values and training targets to the range of 0.15–0.85. Subsequently, the network output is automatically rescaled to the appropriate range.

**Figure 4:**
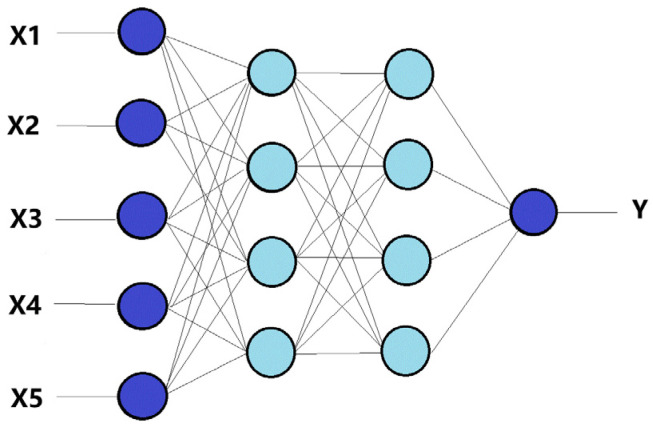
Diagram of the four-layer structure of the ANN-L2 neural network.

After conducting a series of tests, a network with a 5-[4-4]-1 structure was developed (Figure 4).

**Figure 5:**
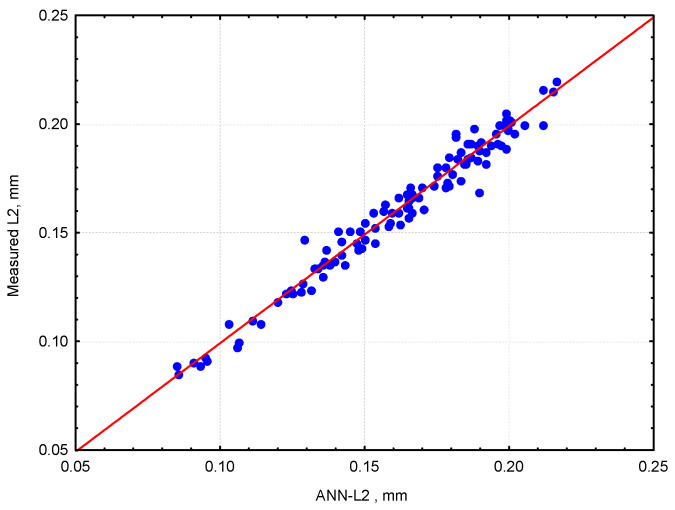
Measured spacing factor L2 versus ANN-L2 outputs.

A comparison between the measured spacing factor L2 and the values determined using the ANN-L2 network is illustrated in Figure 5.

The precision of the estimation related to the training and testing of the data set is presented in Table 4. A comparison of the relationship determined using the 2D Curve program is shown in Figure 5. After rejecting four outliers exceeding 2·Sd, the error is 0.00457. Therefore, it can be stated that there is a very strong correlation between the analyzed input factors and the spacing factor (L2) value. This allows for the calculation of the spacing factor (L2) value with an accuracy of ±2 · 0.00457 = 0.0091.

**Figure A1:**
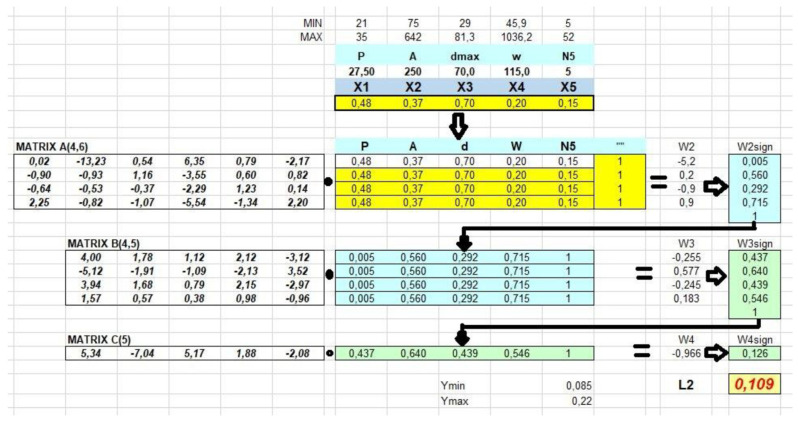
The calculation scheme performed in an Excel spreadsheet according to the ANN-L2 network.

As a result of the calculations performed using the QNET program, the model ANN-L2 = f (P, A, d, W, N5) was obtained, representing a certain form of regression relationship. Basic information regarding the operation of the given artificial neural network is contained in the weight matrices. The relevant matrices (denoted as MATRIX A(), MATRIX B(), and MATRIX C()) for the ANN-L2 model are presented in Appendix A. By knowing these matrices and the method of normalizing input/output data, calculations can be performed in an Excel spreadsheet without the need for specialized software such as QNET and STATISTICA. The diagram illustrating the calculation method of the spacing factor (L2) is shown in Figure A1 (see Appendix A).

## 4. Analysis of the ANN-L2 Model

The quality of air-entrainment depends on the parameters (A, d, W) that describe the chord size distribution. Other factors affecting concrete air-entrainment are the composition-dependent parameter (P) and the number of large pores (chords) (N5), which is influenced by the compaction of the mix.

**Figure 6:**
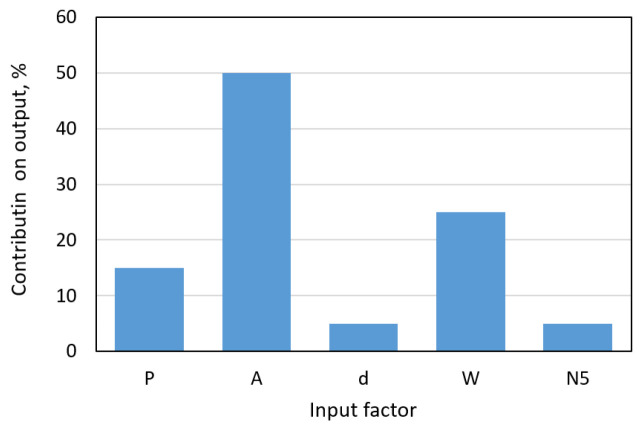
Contributions of the inputs to the output of the ANN-L2 neural network.

As shown in Figure 6, the most significant impact on the spacing factor (L2) is attributed to the peak amplitude (A), peak width (W), and cement paste content (P). In contrast the influences of the chord number (N5) and chord length (d) are considerably smaller.

**Figure 7:**
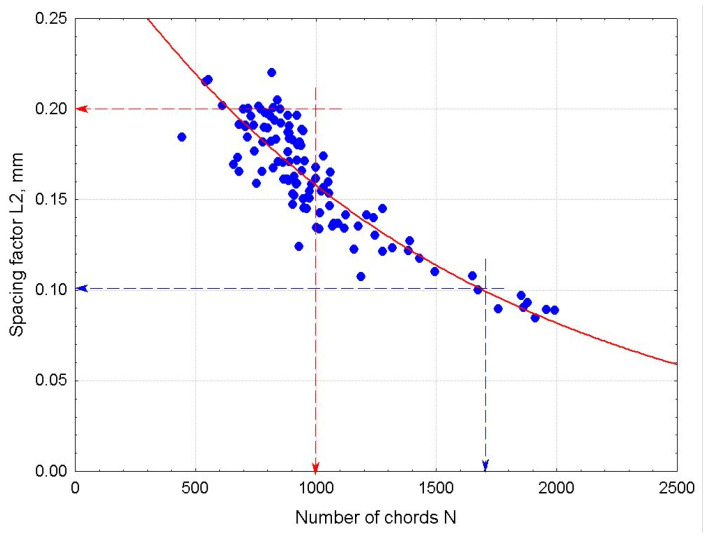
Dependence of the spacing factor (L2) on the total number of chords (N).

**Figure 8:**
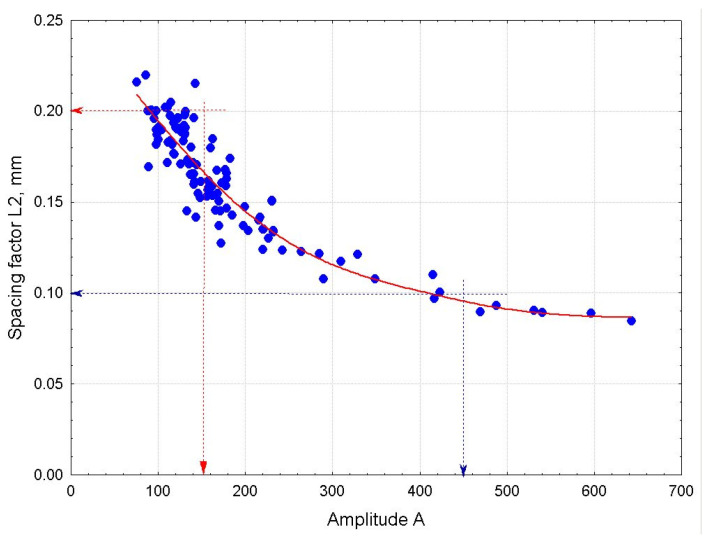
Dependence of the spacing factor (L2) on the peak amplitude (A).

The amplitude (A) and peak width (W), which define the area under the distribution curve, are, therefore, directly related to the total number of recorded chords (N) in a given concrete mix. The strong correlation between the spacing factor (L2) and the total number of chords (N) is illustrated in Figure 7. A very similar relationship between the spacing factor (L2) and the peak amplitude (A) is shown in Figure 8.

From the analysis of the collected data set, it follows that a recorded chord count of N > 1000 guarantees that the spacing factor (L2) is less than 0.20 mm. This condition is also met when the peak amplitude (A) exceeds 150. On the other hand, a high chord (or pore) count of N > 1600 or a peak amplitude (A) greater than 400 indicates that the spacing factor (L2) is below 0.10 mm. Further increases in the chord count have little effect on reducing the spacing factor’s (L2) value; instead, they lead to an unnecessary rise in the overall air content and, consequently, a reduction in concrete strength.

**Figure 9:**
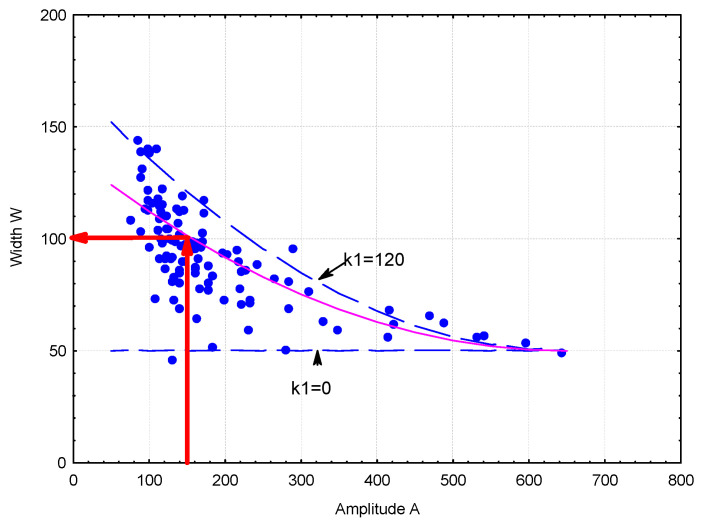
Method for determining the width’s (W) value relative to the amplitude (A).

An analysis of the ANN-L2 model was performed to evaluate the effect of input parameters on the spacing factor (L2). This task is not simple due to the five input factors. When performing calculations, it should be noted that the model was developed for the specific ranges of variability of these factors (domain). The input parameter values should be taken from the appropriate ranges. The adopted method for calculating the value of L2 based on the chord size distribution begins by assuming the amplitude’s (A) value. The peak width (W) can vary over a wide range (see Figure 9).

Assuming an additional coefficient (k1) with values in the range 0 ÷ 120, the value of (W) is calculated using Expression (1).

(1) $$W=50+k1*{\left(\frac{650-A}{650}\right)}^{2},k1=<0,120>$$

(2) d=10+0.43∗W−0.125∗N5+k2;k2=<0,12>

**Figure 10:**
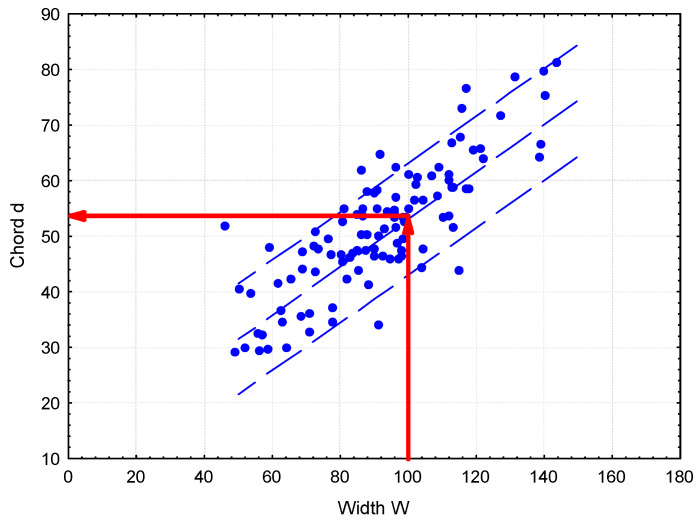
Method for determining the chord’s (d) value in relation to the peak width (W) and the number of large chords (N5).

Subsequently, for a given value of W, the index (d) is calculated using Formula (2). As shown in Figure 10, the value of the index (d) (representing the chord corresponding to the maximum of the chord distribution) depends on both the width (W) and the number of large chords (N5). The index (d) can be adjusted to a certain extent by varying the coefficient (k2) within the range 0–12.

**Figure 11:**
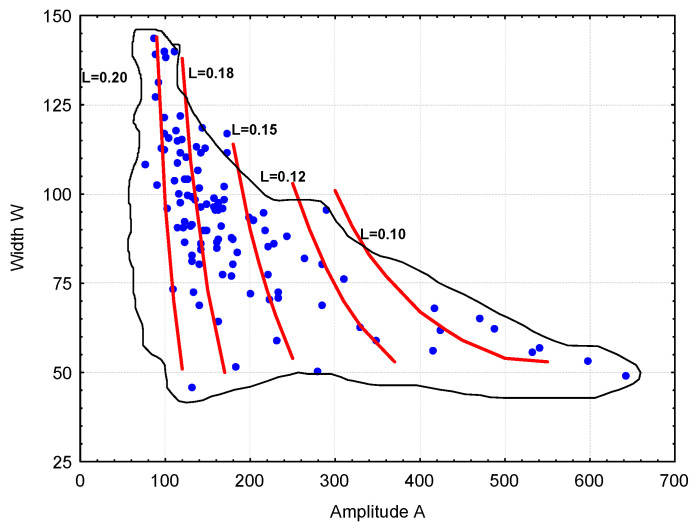
Dependence of the spacing factor (L2) on the peak amplitude (A) and peak width (W) (*p* = 28.5%, N5 = 25).

A series of calculations were conducted to demonstrate the relationship between the spacing factor (L2) and both the amplitude (A) and the peak width (W), as shown in Figure 11. It follows that, for a given amplitude (A), an increase in the peak width (W) results in a decrease in the spacing factor (L2). This effect is more pronounced at higher amplitude (A) values.

## 5. Conclusions

The objective of this study was to determine whether analyzing the chord length distribution can provide a more accurate estimation of the spacing factor. To achieve this, an attempt was made to correlate the standard spacing factor with a model-derived value, incorporating key parameters associated with the chord length distribution.

The artificial neural network (ANN) method was applied as a technique for modeling complex multifactor regression problems. As a result, the ANN-L2 model was developed, representing an implicit nonlinear “black box” function. A data set containing 110 air-entrained concretes with diverse characteristics was used for training and testing the neural network. The results show that the ANN-L2 model enables a sufficiently accurate estimation of the spacing factor (L2). The most significant impact on L2 is attributed to the following:

- Peak amplitude (A);
- Peak width (W);
- Cement paste content (P).

The influence of the number of chords in the 500 ÷ 1000 µm range (N5) and chord length (d) is considerably smaller.

The strong correlation between the ANN-L2 model results and the standard spacing factor (L2) indicates that both calculation methods yield comparable results. However, estimation accuracy issues arise when the number of chords is low or when a significant number of large pores are present in the analyzed concrete.

Therefore, it is difficult to state unequivocally that taking into account the chord distribution parameters in the analysis provides clear benefits. Since the procedure for determining the chord distribution parameters is more labor-intensive, it does not constitute a competing alternative to the standard calculation method. Instead, greater attention should likely be given to ensuring a sufficient number of chords to achieve adequate reliability of the overall analysis.

In recent years, there has been growing interest in automated image analysis (AIA) and 2D techniques for determining air-void structure parameters. This approach offers several potential advantages, as follows:

- A significantly larger number of pores can be analyzed;
- Evaluating pore diameters instead of chord lengths may allow for eliminating large-sized pores from the analysis, improving the pore size distribution accuracy and enhancing the spacing factor estimation.

Considering these aspects, further studies are planned using the presented approach to verify whether an analysis of the pore diameter distribution from measurements (2D) can provide better results than the standard spacing factor.

## Figures and Tables

**Table 1:** Air–void parameter equations.

Parameter	Air Content, %	Specific Surface, mm^−1^	Spacing Factor, mm
Equations	A = (T_a_/T_t_)·100	α = 4N/T_a_	L = 3/α [1.4 (1 + P/A)^1/3^ − 1] when P/A > 4.342
			L = P/(αA) when P/A ≤ 4.342

P is the paste content; A is the air content; α is the specific surface; T_a_ is the length of the chords; T_t_ is the total length of the measuring lines for two samples; N is the number of chord lines for two samples; and N is the number of chords.

**Table 2:** Example of a table with the number of chords across the selected 25 classes.

Class	Number	Number %	Cumulative	Cumulative %
[0; 20)	37	9.16	37	9.16
[20; 40)	107	26.49	144	35.64
[40; 60)	75	18.56	219	54.21
[60; 80)	54	13.37	273	67.57
[80; 100)	31	7.67	304	75.25
[100; 120)	30	7.43	334	82.67
[120; 140)	17	4.21	351	86.88
[140; 160)	14	3.47	365	90.35
[160; 180)	10	2.48	375	92.82
[180; 200)	5	1.24	380	94.06
[200; 220)	2	0.5	382	94.55
[220; 240)	4	0.99	386	95.54
[240; 260)	2	0.5	388	96.04
[260; 280)	3	0.74	391	96.78
[280; 300)	3	0.74	394	97.52
[300; 320)	2	0.5	396	98.02
[320; 340)	0	0	396	98.02
[340; 360)	1	0.25	397	98.27
[360; 380)	1	0.25	398	98.51
[380; 400)	0	0	398	98.51
[400; 420)	1	0.25	399	98.76
[420; 440)	0	0	399	98.76
[440; 460)	2	0.5	401	99.26
[460; 480)	1	0.25	402	99.5
[480; 500]	2	0.5	404	100

**Table 3:** Information characterizing the variability in the parameters related to the composition and distribution of individual concretes.

Factor	P	A	d	W	N5	L2
Input/Output	X1	X2	X3	X4	X5	Y
Mean	29.6	186.6	51.5	100.8	20.5	0.161
Median	28.5	148.0	51.6	92.6	20.0	0.166
Sd	10.0	111.8	11.5	93.0	8.7	0.032
Min	21.0	75.4	29.0	45.9	5.0	0.085
Max	28.5	642.3	81.3	1036.2	52.0	0.220

Legend: P—paste content in concrete (%), A, d, W—chord distribution parameters, N5—number of chords recorded in the range 500 – 1000 µm, L2—spacing factor calculated considering chords up to 2000 µm.

**Table 4:** Estimation of the ANN-L2 model errors.

	N	RMS Error	Correlation
Training set	90	0.0181	0.994
Test set	20	0.0137	0.956

## Data Availability

Data are contained within the article.

## Appendix A

As a result of the calculations performed using the QNET program, the model ANN-L2 = f(P, A, d, W, N5) was obtained, representing a certain form of regression relationship. Basic information regarding the operation of the given artificial neural network is contained in the weight matrices. The relevant matrices (denoted as MATRIX A(), MATRIX B(), and MATRIX C()) for the ANN-L2 model are presented in Figure A1. By knowing these matrices and the method of normalizing the input/output data, calculations can be performed in an Excel spreadsheet without the need for specialized software such as QNET and STATISTICA. The diagram illustrating the calculation method for the spacing factor (L2) is shown in Figure A1.
